# An Integrative Approach to Treat Parkinson's Disease: Ukgansan Complements L-Dopa by Ameliorating Dopaminergic Neuronal Damage and L-Dopa-Induced Dyskinesia in Mice

**DOI:** 10.3389/fnagi.2018.00431

**Published:** 2019-01-07

**Authors:** Eugene Huh, Jin Gyu Choi, Yeomoon Sim, Myung Sook Oh

**Affiliations:** ^1^Department of Medical Science of Meridian, Graduate School, Kyung Hee University, Seoul, South Korea; ^2^Department of Life and Nanopharmaceutical Sciences, Graduate School, Kyung Hee University, Seoul, South Korea; ^3^BK21 PLUS Integrated Education and Research Center for Nature-inspired Drug Development Targeting Healthy Aging, Kyung Hee University, Seoul, South Korea; ^4^Department of Oriental Pharmaceutical Science, College of Pharmacy, Kyung Hee East-West Pharmaceutical Research Institute, Kyung Hee University, Seoul, South Korea

**Keywords:** Parkinson's disease, levodopa, ukgansan, levodopa-induced dyskinesia, 6-hydroxydopamine

## Abstract

Parkinson's disease (PD) is accompanied by motor impairments due to the loss of dopaminergic neurons in the nigrostriatal pathway. Levodopa (L-dopa) has been the gold standard therapy for PD since the 1960s; however, its neurotoxic features accelerate PD progression through auto-oxidation or the induction of dyskinetic movements. Ukgansan (UGS) is a well-known prescription for treating PD in traditional medicines of East Asia, and its anti-PD function has been experimentally evaluated. The present study investigated whether UGS attenuates (1) motor dysfunction and dopaminergic neuronal damage when co-treated with L-dopa and (2) L-dopa-induced dyskinesia (LID) in 6-hydroxydopamine (6-OHDA)-induced PD mice. Although L-dopa was found to reduce motor dysfunctions, it failed to decrease the dopaminergic neuronal damage and increased the expression of dopamine receptor 1 (D1R) and 2 (D2R) in the 6-OHDA-injected mouse striatum. Co-treatment with UGS resulted in normal striatal histology and ameliorated motor impairments. In addition, UGS suppressed the dyskinesia induced by chronic L-dopa treatment while restoring the dopaminergic neurons in the striatum. For the underlying mechanism, UGS reduced the overexpression of D1R-related signaling proteins, such as phosphorylated extracellular signal-regulated kinase, ΔFosB, and c-fos in the striatum. Overall, the results suggest that the effect of UGS could be complementary to L-dopa by ameliorating motor dysfunction, restoring the dopaminergic neurons, and suppressing the dyskinetic movements in PD.

## Introduction

Parkinson's disease (PD), one of the most typical neurodegenerative diseases, accounts for 1–2% of the population over 65 years of age, presenting motor dysfunctions such as bradykinesia, tremor, rigidity, and postural instability (Jankovic, 2008). These manifestations are caused by the lack of dopamine via loss of dopaminergic neurons in the substantia nigra pars compacta (SNpc) and the striatum (ST) (De Lau and Breteler, 2006). Most current treatments for PD mainly rely on dopaminergic substitution for the purpose of relieving symptoms (Schapira et al., 2014).

Levodopa (L-dopa), a precursor of dopamine, is the gold standard therapy for treating symptoms of PD via supplying exogenous dopamine, which reverses the distinctive behavioral indications of PD. However, there are some critical limitations of L-dopa since its effects are merely symptomatic and not as fundamental. One such limitation is that L-dopa may accelerate dopaminergic neuronal death during the progression of PD (Fahn et al., 2004). In addition, chronic treatment with L-dopa is accompanied by severe side effects that are induced by failure to control the concentration of exogenous dopamine. Up to90% of PD patients treated by L-dopa administration for 9–10 years have experienced L-dopa-induced dyskinesia (LID), which is the chief complaint (Connolly and Lang, 2014). LID involves abnormal involuntary movements (AIM) that represent a clinical therapeutic problem and worsen patients' quality of life. Though the mechanisms of LID have not been clearly revealed, it is closely related with the hyperactivation of the dopamine receptor 1 (D1R)-related signaling pathway in striatal projection neurons (Feyder et al., 2011; Fieblinger et al., 2014).

Herbal medicines have been widely utilized as traditional medicines since thousands of years. Their use is a promising approach to treating neurodegenerative diseases as they contain a variety of compounds that have a wider range of targets than conventional small-molecule drugs (Koehn and Carter, 2005; Choi et al., 2018). In a systematic approach, Kim et al. (2015) revealed that compounds derived from herbal medicines more closely resemble human metabolites than synthetic compounds. This suggests that herbal medicines may be more suitable for treating disease than conventional drugs and may offer an integrative approach required to treat multifactorial diseases such as PD.

Ukgansan (UGS) is composed of seven medicinal herbs and is the most used prescription for neurodegenerative diseases such as dementia and PD in traditional medicines of East Asia (Iwasaki et al., 2005). In PD patients, treatment with UGS for 4 weeks improved behavioral and psychological symptoms via regulating various neurotransmissions (Kawanabe et al., 2010). Similarly, 25 PD patients treated with UGS for 12 weeks showed significant reduction in neuropsychiatric symptoms (Hatano et al., 2014). UGS has been shown to reduce parkinsonism symptoms in drug-induced PD patients (Shim et al., 2015). Doo et al. (2010) revealed the neuroprotective effects of UGS against 1-methyl-4-phenyl-1,2,3,6-tetrahydropyridene (MPTP) neurotoxicity via upregulating PI3K/Akt pathway. In addition, it has been reported that UGS inhibits catechol-O-methyltransferase (COMT) to assist dopamine supplementation (Ishida et al., 2016). However, it is unclear whether UGS could support the effects of L-dopa on motor dysfunctions or suppress the hyperkinetic movements accompanied by chronic treatment with L-dopa in a PD mouse model.

This study aimed to investigate whether (1) UGS and L-dopa ameliorate motor dysfunction and dopaminergic neuronal damage induced by 6-hydroxydopamine (6-OHDA) when treated concurrently, and whether (2) UGS reduces LID and its related histological changes. To investigate the effects of UGS and L-dopa on 6-OHDA-induced motor impairments and dopaminergic neuronal loss, we performed the rotarod and apomorphine-induced rotation tests, and histological analysis of the ST and SNpc. To investigate the effect of UGS on dyskinetic movements in 6-OHDA-injected mice treated with L-dopa chronically, we measured the score of AIM scale (AIMs) and analyzed the concomitant histological changes in the ST.

## Materials and Methods

### Animals

Animal maintenance and treatments were performed in accordance with the Animal Care and Use Guidelines of Kyung Hee University, Seoul, Korea (approved number; KHUASP(SE)-17-020). Male ICR mice (6 weeks old, 30–32 g) were purchased from the Daehan Biolink Co., Ltd. (Eumseong, Korea). The animals were housed 4 per cage (size 40 × 25 × 18 cm) with free access to water and food and were kept under constant temperature (23 ± 1°C) and humidity (60 ± 10%) and a 12-h light/dark cycle. Mice were adapted to their surroundings for 7 days and kept under the same conditions before the start of the study.

### Surgery Procedure

Mice were anesthetized with tribromoethanol (312.5 mg/kg, i.p.) (Salazar et al., 2010) and placed on a stereotaxic apparatus (myNeuroLab, St. Louis, MO, USA). Each mouse received a unilateral injection of 2 μL vehicle (saline with 0.1% ascorbic acid, for sham-operated mice) or 6-OHDA (8 μg/μL) into the right ST (coordinates with respect to bregma in mm: AP 0.5, ML 2.0, DV−3.0), according to the stereotaxic atlas of mouse brain (Franklin and Paxinos, 2013). 6-OHDA solution was delivered by a microinjection pump at an injection rate of 0.5 μL/min, and the cannula was left in place for 4 min after the end of injection. After surgery, mice were allowed to recover from anesthesia in a temperature-controlled chamber and then placed in individual cages.

### Preparation of UGS

UGS is composed of seven dried medicinal herbs: Atractylodes lancea rhizome (4.0 g, rhizome of *Atractylodes lancea* De Candolle), Poria sclerotium (4.0 g, sclerotium of *Poria cocos* Wolf), Cnidium rhizome (3.0 g, rhizome of *Cnidium officinale* Makino), Uncaria Hook (3.0 g, thorn of *Uncaria rhynchophylla* Miquel), Japanese Angelica Root (3.0 g, root of *Angelica acutiloba* Kitagawa), Bupleurum Root (2.0 g, root of *Bupleurum falcatum* Linne), and Glycyrrhiza (1.5 g, root and stolon of *Glycyrrhiza uralensis* Fisher). It was supplied by Tsumura & Co. (Tokyo, Japan) as a dry, powdered extract. Each plant used in the preparation of UGS was identified by its external morphology and authenticated against known specimens according to the methods of the Japanese Pharmacopoeia and the company's standards, previously reported (Mizukami et al., 2009).

### Drug Administration

**Figure 1:**
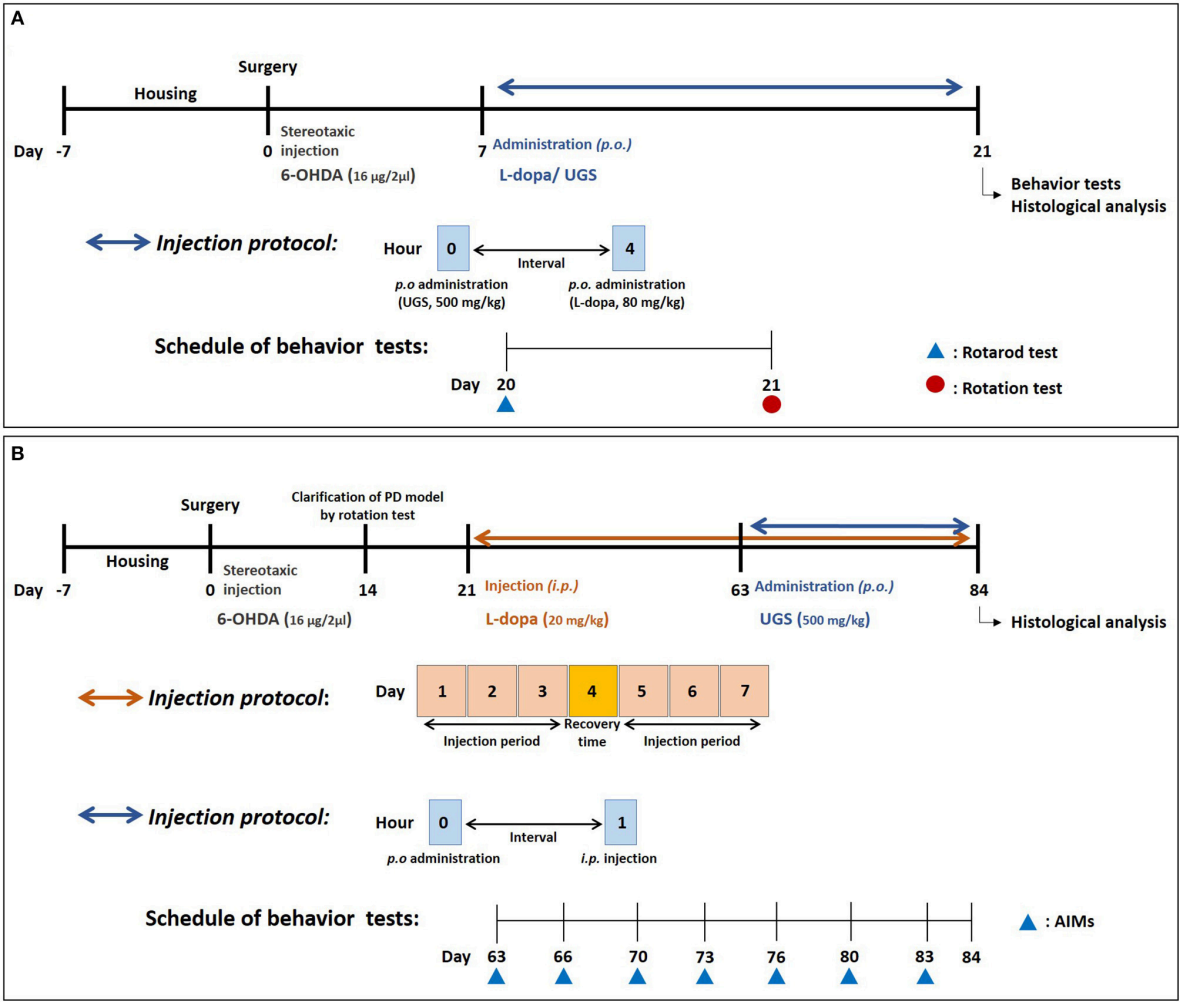
Experimental schedule. The schedule for investigating the effects of UGS and L-dopa on motor dysfunctions **(A)** and for the effects of UGS on LID **(B)**.

As shown in Figure 1A, drugs were administered once daily for 14 days starting from the 7^th^ day after stereotaxic injection. The 56 animals used in the study were divided into 7 groups with 8 mice in each group: (1) Sham group (Sham-operated plus oral vehicle treatment), (2) L-dopa group (Sham-operated plus oral L-dopa treatment [80 mg/kg/day for 14 days]), (3) UGS group (Sham-operated plus oral UGS treatment [500 mg/kg/day for 14 days]), (4) 6-OHDA group (6-OHDA-lesioned plus oral vehicle treatment); (5) 6-OHDA + L-dopa group (6-OHDA-lesioned plus oral L-dopa treatment [80 mg/kg/day for 14 days]), (6) 6-OHDA + UGS group (6-OHDA-lesioned plus oral UGS treatment [500 mg/kg/day for 14 days]), and (7) 6-OHDA + L-dopa + UGS group (6-OHDA-lesioned plus oral L-dopa and UGS treatment [80 mg/kg/day and 500 mg/kg/day, respectively, for 14 days]). L-dopa (Sigma-Aldrich, MO, USA) and UGS were dissolved in normal saline. The administration of UGS was conducted at least 4 h after the administration of L-dopa. Equal volumes of the vehicle were administered to sham and 6-OHDA mice.

The scheme for investigating the effect of UGS on LID was as follows (Figure 1B): on the 14th day post-surgery, the rotation test was performed to assess coordination in the PD model. L-dopa (20 mg/kg with 10 mg/kg benserazide) was administered for 42 days starting on the 21st day after stereotaxic injection, reflecting the clinical period of L-dopa administration. The administration schedule was as follows: 3 days for injection and 1 day for recovery. After the development of LID, 32 animals were divided into 4 groups with 8 mice in each group: (1) Sham group (Sham-operated plus intraperitoneal vehicle treatment), (2) PD group (6-OHDA-lesioned plus intraperitoneal vehicle treatment), (3) LID group (6-OHDA-lesioned plus intraperitoneal L-dopa treatment [20 mg/kg/day for 21 days]), and (4) LID + UGS (6-OHDA-lesioned plus intraperitoneal L-dopa treatment [20 mg/kg/day for 21 days] and oral UGS treatment [500 mg/kg/day for 21 days]) where L-dopa and UGS were dissolved in normal saline.

### Behavioral Tests

#### Rotarod Test

To measure the bradykinesia and hypokinesia induced by 6-OHDA in mouse, we performed the rotarod test. The rotarod unit was made of a rotating spindle (7.3 cm diameter) and five individual compartments able to simultaneously test five mice. After two successive days of twice-daily training (4 rpm rotation speed on the first day and 12 rpm on the second day), the test rotation speed was increased to 14 rpm on the last day in a test session. The time each mouse remained on the rotating bar was recorded over three trials per mouse, at 5 min intervals and the maximum test time was limited up to 180 s. Data were shown as the mean time on the rotating bar over the three test trials.

#### Apomorphine-Induced Rotation Test

The mice were placed in hemispheric rotational bowl with a diameter of 40 cm. They were allowed to habituate to their environment for 5 min before the administration of apomorphine (4 mg/kg, *s.c*.). Full 360° turns in the direction opposite to the lesion (contralateral rotation) were counted and rotational behaviors were assessed for 25 min. Results were expressed as contralateral turns in 25 min.

#### AIMs Scoring Test

To evaluate the dyskinesia induced by L-dopa, AIMs scoring tests were performed and modified based on previous studies (Pavon et al., 2006; Dos-Santos-Pereira et al., 2016). A trained observer assessed each mouse for the presence of AIMs at 20-min intervals for 180 min after L-dopa administration. The assessment took place on days 0, 3, 7, 10, 13, 17, and 20 following the development of the LID model. Axial, limb, and orolingual AIMs subtypes were scored in 1-min periods. Each subtype was measured accordingly on a severity scale ranging from 0 to 4 (0 = absent, 1 = occasional, 2 = frequent, 3 = continuous but interrupted by sensory distraction, and 4 = continuous, severe, and insuppressible).

### Western Blot Analysis

Brain tissues were lysed using a protein assay kit according to the manufacturer's instructions. Protein concentrations in each fraction were quantified by Bradford's assay by using kit according to the manufacture's instruction (Bio-rad, Hercules, CA, USA). Equal amount of protein in each sample was separated on 10 % sodium dodecyl sulfate–polyacrylamide gel electrophoresis, and then separated proteins were electrophoretically transferred to a membrane. The membranes were blocked with 5% skim milk in 25 mM tris-Cl, 150 mM NaCl, and 0.005% Tween-20 for 45 min. Thereafter, the membranes were incubated overnight with primary antibodies at 4°C. The primary antibodies were directed against D1R, dopamine receptor 2 (D2R) (Abcam, MA, USA), FosB, c-fos, extracellular signal-regulated kinase (ERK), phosphorylated ERK (p-ERK), and β-actin (Santa Cruz, CA, USA). The blots were washed three times for 10 min with tris-buffered saline with tween 20 (TBS-T). Then, the membranes were incubated with respective horseradish peroxidase-conjugated secondary antibodies for 1 h and washed with TBS-T again. Immunoreactive bands were developed using an enzyme-linked chemiluminescence detection kit and visualized using the ChemiDoc^TM^ XRS+ system (Bio-rad, CA, USA).

### Immunohistochemistry and Quantification

Mice were anesthetized with tribromoethanol (312.5 mg/kg, *i.p*.), perfused transcardially with 0.05 M phosphate-buffered saline (PBS) and then fixed with cold 4% paraformaldehyde (PFA) in 0.1 M phosphate buffer (PB). Brains were removed and post-fixed in 0.1 M PB containing 4% PFA overnight at 4°C and then immersed in a solution containing 30% sucrose in 0.05 M PBS for cryoprotection. Serial 30 μm-thick coronal sections were cut on a freezing microtome (Leica Instruments GmbH, Nussloch, Germany) and stored in cryoprotectant (25% ethylene glycol, 25% glycerol, and 0.05 M PB) at −20°C until use for immunohistochemistry.

For each mouse, brain regions including ST (at Bregma 0.98 to 0.50 mm Li et al., 2000) and SN [at Bregma −2.92 to −3.52 mm (L'episcopo et al., 2013)] were used for the immunohistochemistry. Cell counting was conducted by an experimenter who did not know the treatment condition, and the result for each animal was the average of the number from its three sections.

Free floating brain sections were rinsed in PBS before immunostaining and then pretreated with 1% hydrogen peroxide for 15 min to remove endogenous peroxidase activity. Then, they were incubated overnight with a rabbit anti-tyrosine hydroxylase (TH) (1:1000 dilution) or anti-dopamine transporter (DAT) antibodies (1:500 dilution) (Abcam, MA, USA) in PBS containing 0.3% Triton X-100 and normal goat serum. They were then incubated with a biotinylated anti-rabbit IgG (1:200 dilution) for 1 h 20 min followed by incubation in avidin-biotin complex solution for 1 h at room temperature. The color of every section was developed with 3,3-diaminobenzidine for 30 sec to 1 min. Quantification of the brain tissue sections was performed by counting the number of TH-immunopositive neurons in the SNpc at × 200 magnification and measuring the optical density of TH-positive and DAT-positive fibers in the ST at × 40 magnification using Image J software (Bethesda, MD, USA). Data were presented as a percent of the contralateral side of each section values. The images were photographed using an optical light microscope (Olympus Microscope System BX51; Olympus, Tokyo, Japan).

### Statistical Analysis

All statistical parameters were calculated using GraphPad Prism 5.01 software (GraphPad Software Inc., San Diego, USA). Values were expressed as the mean ± standard error of the mean (S.E.M.). The results except AIMs scores were analyzed by one-way analysis of variance (ANOVA) followed by Bonferroni's multiple comparison test among all groups. AIMs scores were analyzed by repeated measures of ANOVA with Bonferroni's post hoc tests for pairwise multiple comparisons. Differences with a p value less than 0.05 were considered statistically significant.

## Results

### UGS Attenuated 6-OHDA-Induced Motor Dysfunctions When Co-treated With L-Dopa

**Figure 2:**
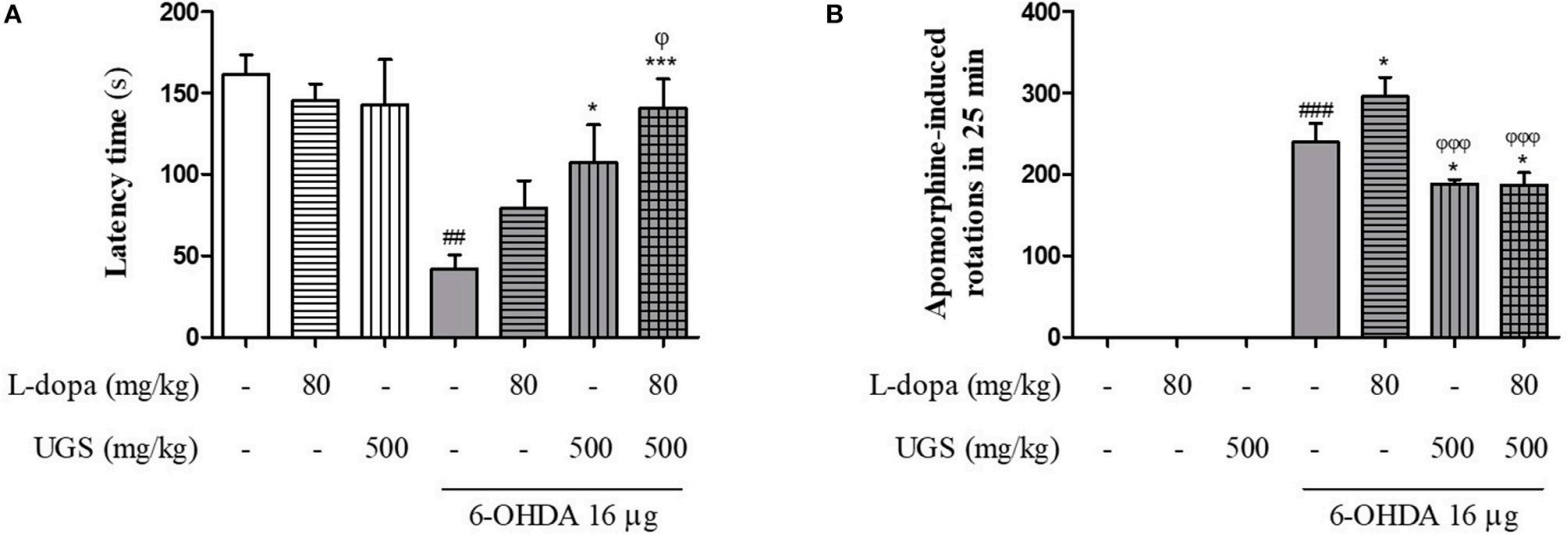
UGS attenuated 6-OHDA-induced motor dysfunctions when co-treated with L-dopa. To evaluate the passive motor function, rotarod test was assessed **(A)**. For hemi-parkinsonian model, it can evaluate the effect of dopamine function via apomorphine-induced rotation tests **(B)**. Values are given as the mean ± S.E.M. (*N* = 8). The significance of differences was analyzed by one-way ANOVA followed by Bonferroni's Multiple Comparison Test: ^*##*^*p* < 0.01 and ^*###*^*p* < 0.001; mean values were significantly different from the sham group. ^*^p < 0.05 and ^***^p < 0.001; mean values were significantly different from the 6-OHDA group. ^ϕ^*p* < 0.05 and ^ϕϕϕ^ *p* < 0.001; mean values were significantly different from the 6-OHDA + L-dopa group.

The rotarod test was performed to compare the effects of combined UGS and L-dopa treatment and L-dopa-only treatment. The latency time of the 6-OHDA group was significantly shortened compared with that of the sham group while those of the 6-OHDA + L-dopa, 6-OHDA + UGS, and 6-OHDA + L-dopa + UGS groups were longer than that of the 6-OHDA group. The latency time of the 6-OHDA + L-dopa + UGS group was significantly increased compared to that of the 6-OHDA + L-dopa group (Figure 2A). The apomorphine-induced rotation test was conducted to investigate the alterations in dopamine sensitivity when treated with L-dopa and UGS concurrently or separately. The number of rotations in the 6-OHDA + L-dopa group was higher than that in the 6-OHDA-lesioned group; however, the 6-OHDA + L-dopa + UGS group showed decreased number of rotations compared to the 6-OHDA group and the 6-OHDA + UGS group (Figure 2B).

### UGS Reduced the Dopaminergic Neuronal Damage Induced by 6-OHDA in the Mouse ST and SNpc When Co-treated With L-Dopa, Reduction did not Occur in the L-Dopa Only Treatment

**Figure 3:**
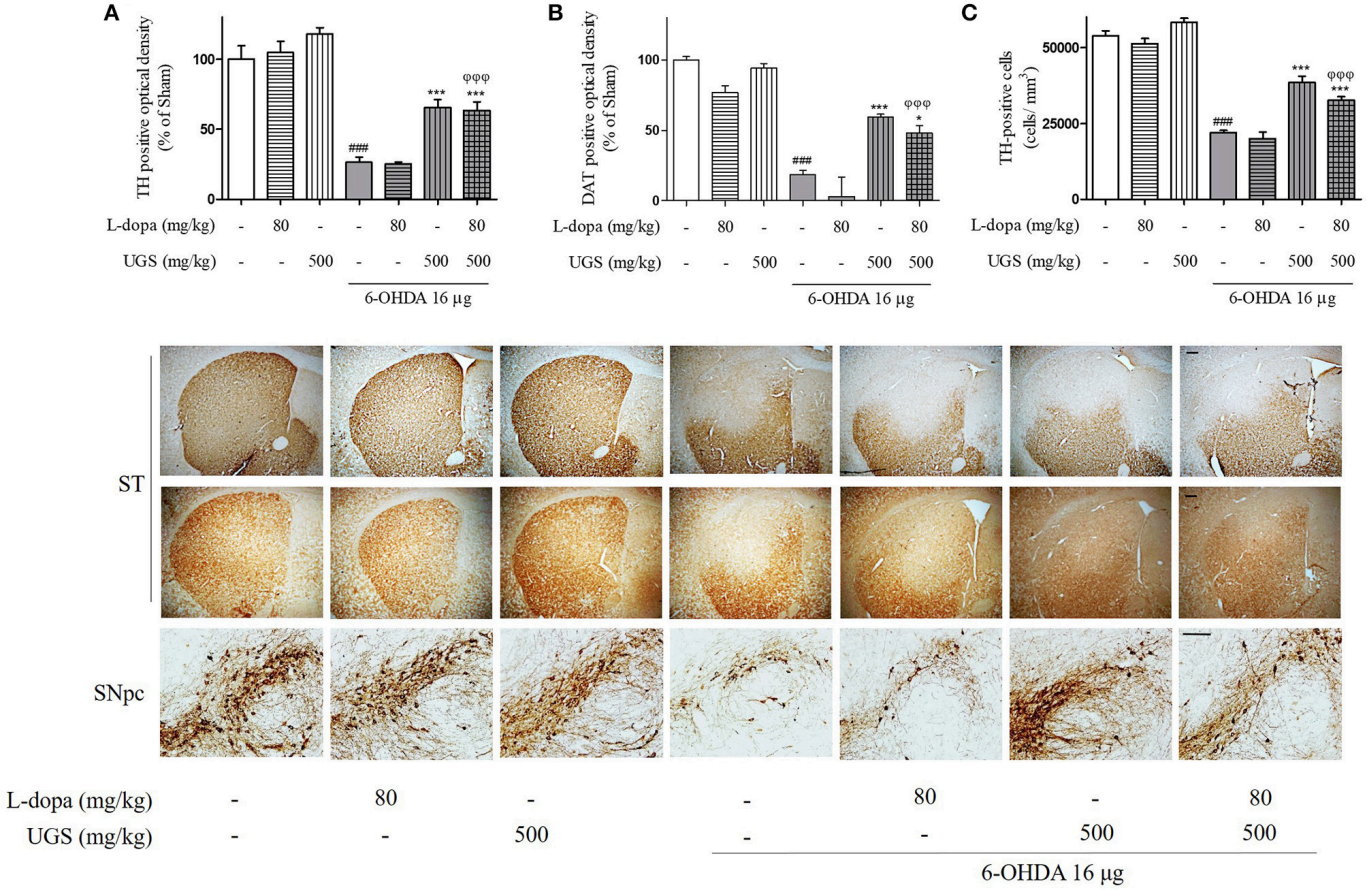
UGS treated with L-dopa reduced the dopaminergic neuronal damage induced by 6-OHDA in the mouse ST and SNpc. To impede the progress of PD, drugs have to restore dopaminergic neurons against toxicity. To evaluate the restorative effect, dopaminergic neurons were visualized with TH and DAT-immunostaining in nigrostriatal pathway. The optical density of TH and DAT-positive fibers in the ST was measured **(A,B)**. The stereological TH-positive neurons in the SNpc were counted **(C)**. Scale bar = 100 μm. Values of quantification data are given as the mean ± S.E.M. (*N* = 8). The significance of differences was analyzed by one-way ANOVA followed by Bonferroni's Multiple Comparison Test: ^###^*p* < 0.001; mean values were significantly different from the sham group. ^*^p < 0.05 and ^***^p < 0.001; mean values were significantly different from the 6-OHDA group. ^ϕϕϕ^*p* < 0.001; mean values were significantly different from the 6-OHDA + L-dopa group.

TH-immunohistochemistry was performed using the SNpc and ST of each mouse brain to test whether co-treatment of UGS with L-dopa reduces 6-OHDA-induced decrease in dopaminergic neuronal cells and fibers in SNpc and ST, respectively. The 6-OHDA group showed significantly decreased optical density of TH-positive fibers in the ST compared to the sham group. The 6-OHDA + UGS and 6-OHDA + L-dopa + UGS groups showed normal optical densities of TH-positive fibers in the ST when compared to the 6-OHDA group. Although the 6-OHDA + L-dopa group showed no improvement in the optical density of TH-positive fibers in the ST, the 6-OHDA + L-dopa + UGS group showed significantly increased TH-positive fibers when compared to the 6-OHDA + L-dopa group (Figure 3A). In the SNpc region, the number of TH-positive neuronal cells of the 6-OHDA group did not differ from that in the 6-OHDA + L-dopa group. However, the 6-OHDA + UGS and 6-OHDA + L-dopa + UGS groups showed significantly reduced dopaminergic neuronal damage. The 6-OHDA + L-dopa + UGS group also showed significantly increased number of TH-positive neuronal cells compared to the 6-OHDA + L-dopa group (Figure 3C).

DAT maintains the concentration of dopamine in the synaptic cleft via the uptake of dopamine into dopaminergic neurons, and its expression indirectly represents the abundance of dopaminergic neurons. To investigate the expression of DAT in the ST, DAT-immunohistochemistry was performed. The expression of DAT was significantly decreased in the 6-OHDA group. Moreover, there were no significant differences in DAT expression between the 6-OHDA + L-dopa group and the 6-OHDA group. However, the 6-OHDA + UGS and 6-OHDA + L-dopa + UGS groups showed significantly increased DAT. Moreover, the 6-OHDA + L-dopa + UGS group showed a higher optical density for DAT than the 6-OHDA + L-dopa group (Figure 3B).

### UGS Reduced the Expression of D1R That Was Increased by L-Dopa in the ST of 6-OHDA Mice

**Figure 4:**
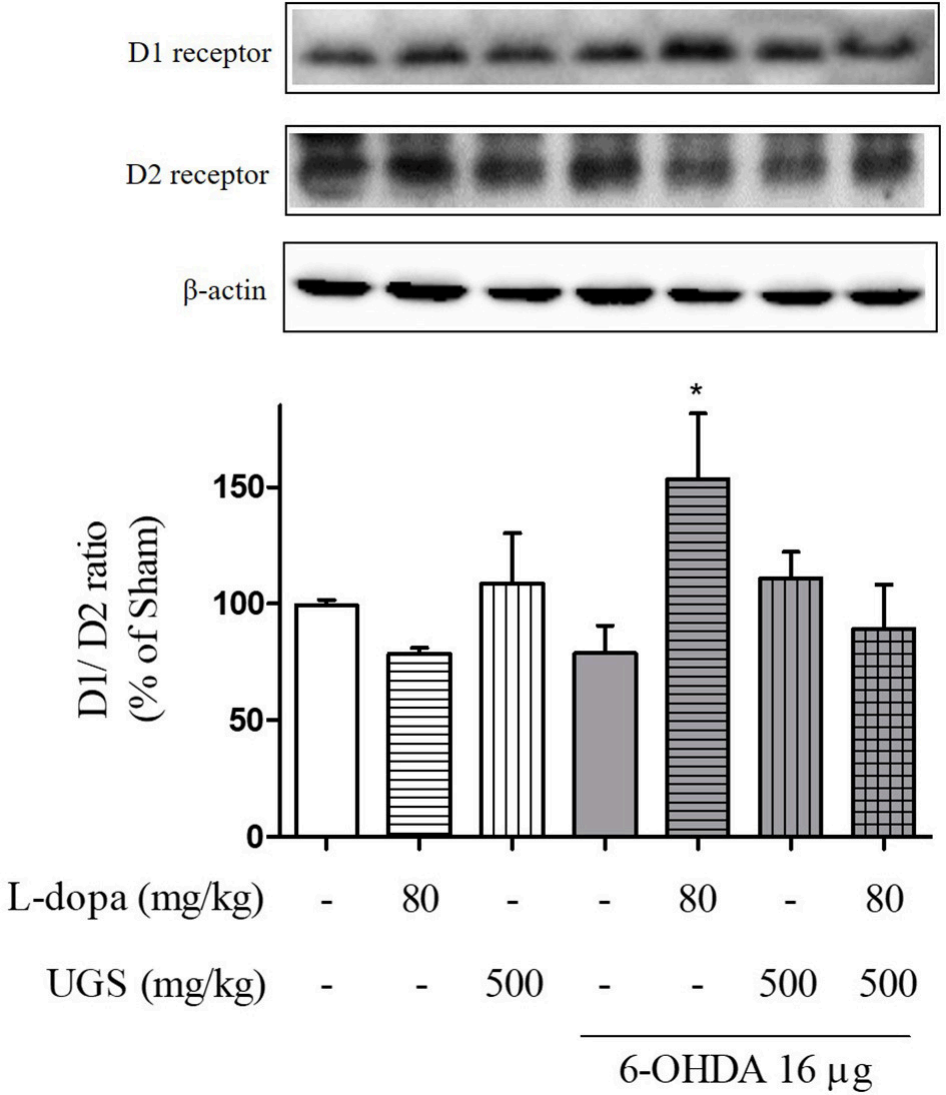
UGS regulated the L-dopa-induced alterations in the expression of dopamine receptors in 6-OHDA-injected mouse ST. Representative band image of the expression of dopamine receptors from western blot analysis, quantification data of D1R and D2R. The D1R/D2R ratio was evaluated. Values of quantification data are given as the mean ± S.E.M. (*N* = 6). The significance of differences was analyzed by one-way ANOVA followed by Bonferroni's Multiple Comparison Test: ^*^p < 0.05; mean values were significantly different from the 6-OHDA group.

L-dopa has been known to induce the upregulation of D1R. Upon chronic use, L-dopa causes an imbalance in dopamine regulatory systems. Therefore, it is important to modulate the activities of dopamine receptors to harmonize the dopaminergic transmission. To investigate the dopaminergic signaling modulation, the western blot was performed to detect whether the co-administration of L-dopa and UGS normalizes the expression of dopamine receptors in the ST. Results showed that the 6-OHDA group had decreased expression of D1R, but increased expression of D2R. Interestingly, the ratio of D1R/D2R was significantly increased in the 6-OHDA + L-dopa group, while the L-dopa group showed opposite results. The 6-OHDA + L-dopa + UGS group showed a normal ratio of D1R and D2R (Figure 4).

We confirmed that the co-administration of UGS with L-dopa significantly ameliorates motor dysfunction in 6-OHDA-injected mice, compared to L-dopa alone. Following this confirmation, we investigated whether UGS had an effect on dyskinesia induced by chronic treatment of L-dopa.

### UGS Suppressed the Dyskinetic Movements in LID Mice

**Figure 5:**
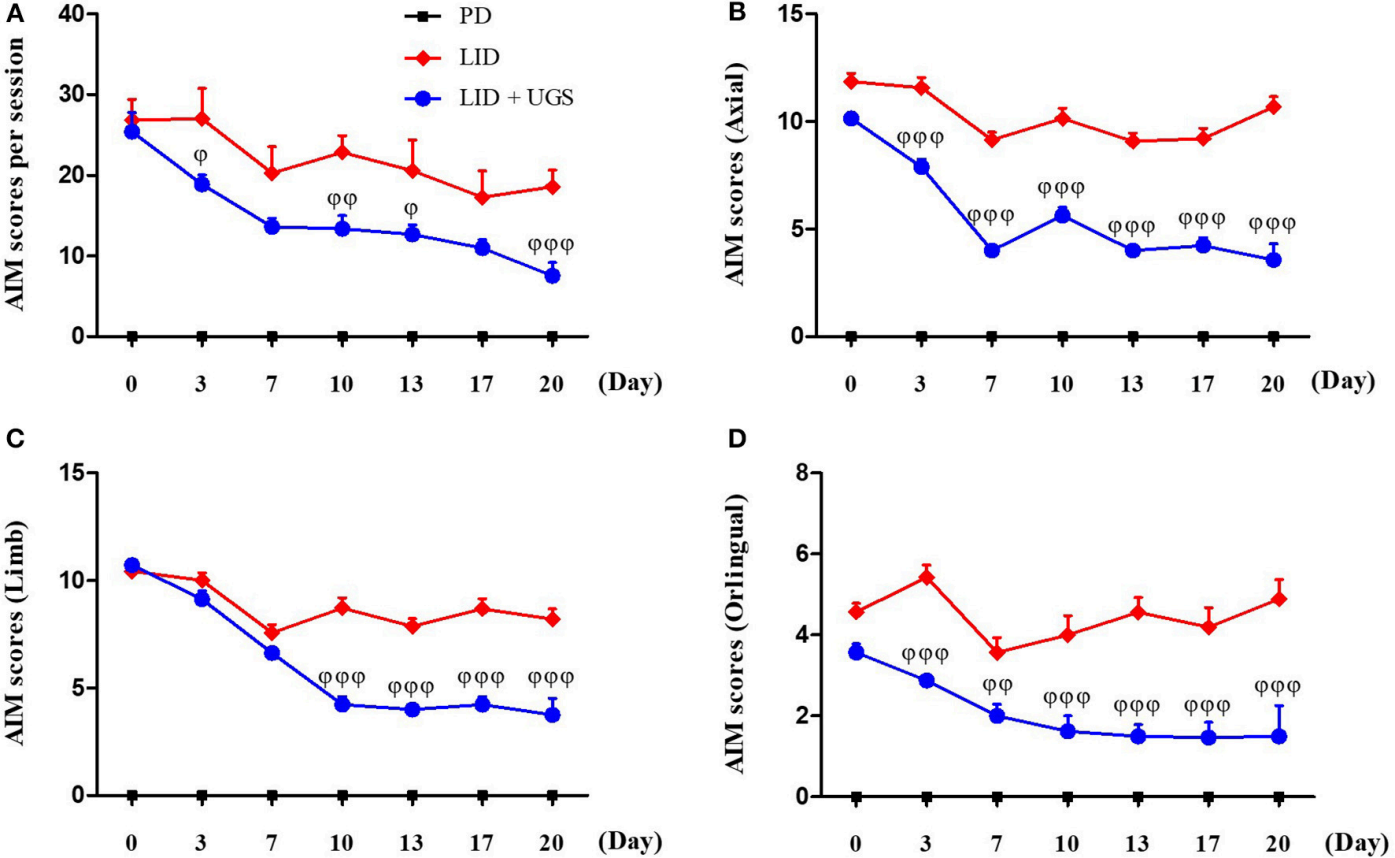
UGS suppressed the dyskinetic movements in LID mice. 6-OHDA-lesioned mice treated with UGS ameliorates LID over the 21 days treatment period, which differed significantly from the LID groups in all testing sessions. The sum of axial, limb, and oroligual **(A)**, axial **(B)**, forelimb **(C)**, and oroligual **(D)** AIMs on each testing session were rated following the administration of UGS. Values are given as the mean ± S.E.M. (*N* = 6). The significance of differences was analyzed by repeated measures of ANOVA with Bonferroni's post hoc tests: ^ϕ^*p* < 0.05, ^ϕϕ^*p* < 0.01, and ^ϕϕϕ^*p* < 0.001; mean values were significantly different from the LID group.

After 42 days of treatment with L-dopa, the PD mice exhibited apparent dyskinetic movements. The scores of axial, limb, and orolingual AIMs assessments had no significant differences between the LID and LID + UGS groups on the first day of assessment. During the 21-day administration period, both the total global and separated (axial, limb, and orolingual) AIMs scores were maintained in the LID group. In the UGS-treated group, all AIMs scores were significantly decreased from the 10th day of administration compared to those in the LID group. Repeated measures of ANOVA revealed a significant anti-dyskinetic effect of UGS [*F*_(3, 168)_ = 12.728, *p* < 0.001] and a significant group^*^time interaction [*F*_(9, 168)_ = 6.301, *p* < 0.001]. The dyskinetic behavior was not seen in the PD group (Figure 5).

**Figure 6:**
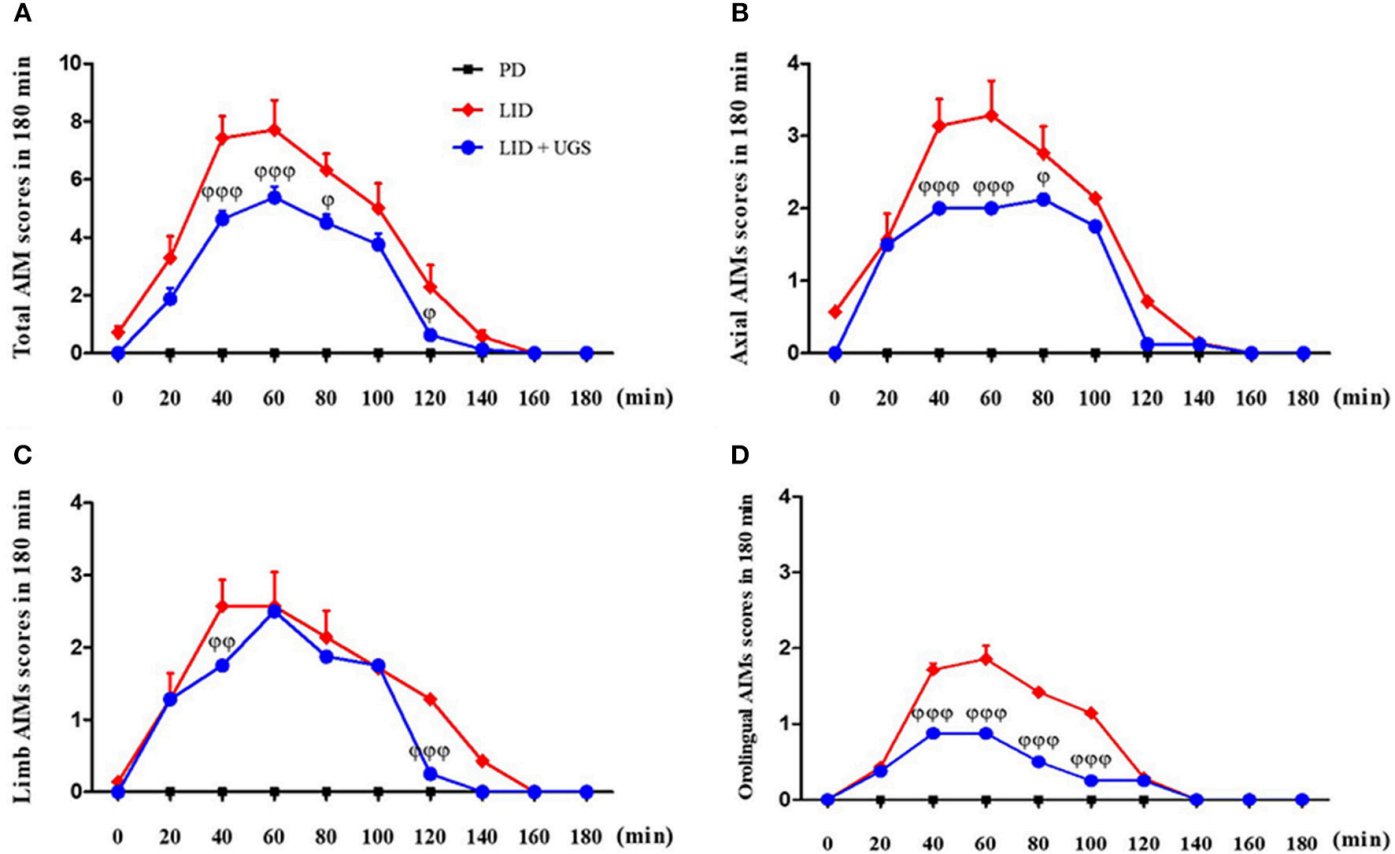
UGS decreased the peak score of LID during 180 min after L-dopa injection. All testing sessions were performed at the 10th day of L-dopa treatment. The sum of axial, limb, and oroligual **(A)**, axial **(B)**, forelimb **(C)**, and oroligual **(D)** AIMs on each testing session were rated every 20 min. There is no significant difference of interaction time as treating UGS in LID mouse. Values are given as the mean ± S.E.M. (*N* = 6). The significance of differences was analyzed by repeated measures of ANOVA with Bonferroni's *post hoc* tests: ^ϕ^*p* < 0.05, ^ϕϕ^*p* < 0.01, and ^ϕϕϕ^*p* < 0.001; mean values were significantly different from the LID group.

To investigate the interaction between UGS and time after treatment with L-dopa, AIMs scores were measured on the 10th day of administration for 180 min after L-dopa injection. In the LID group, all AIMs scores peaked at 60 min after L-dopa injection. Mice treated with UGS showed significantly decreased dyskinetic movements at the peak time compared to LID-induced mice. Mice in both the LID and LID+UGS groups did not show dyskinetic movements 160 min after LID injection. Repeated measures of ANOVA was performed and revealed a significant main effect of UGS [*F*_(3, 245)_ = 25.693, *p* < 0.001] and, a non-significant group^*^time interaction [*F*_(9, 245)_ = 0.735, *p* = 0.636] (Figure 6).

### UGS Attenuated the Dopaminergic Neuronal Loss in the ST of LID Mice

**Figure 7:**
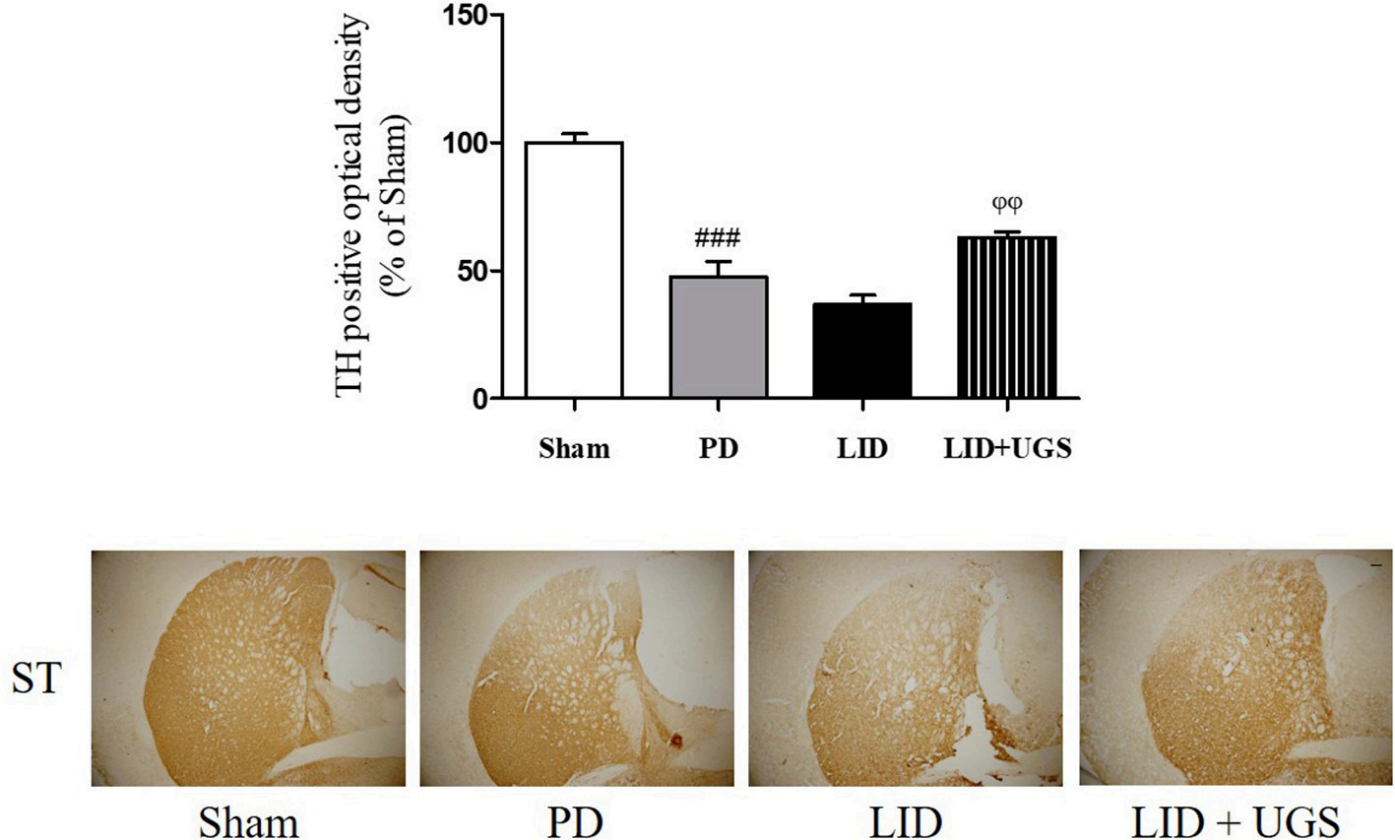
UGS attenuated the dopaminergic neuronal loss in L-dopa chronically treated the mouse ST. The optical density of TH fibers in the ST was measured. Scale bar = 100 μm. Values of quantification data were given as the mean ± S.E.M. (*N* = 6). The significance of differences was analyzed by one-way ANOVA followed by Bonferroni's Multiple Comparison Test: ^###^*p* < 0.001; mean values were significantly different from the sham group. ^ϕϕ^*p* < 0.01; mean values were significantly different from the LID group.

TH-immunohistochemistry in the ST of each mouse brain was performed to detect whether UGS reduced the dopaminergic neuronal damage in the ST of LID mice. The PD and LID groups showed significantly decreased optical density of TH-positive fibers in the ST compared to the sham group. The LID + UGS group showed normal optical densities of TH-positive fibers in the ST, which showed a tendency to be worse in the LID group (Figure 7).

### UGS Normalized the Overexpression of D1R-Related Transmissions in the ST of LID Mice

**Figure 8:**
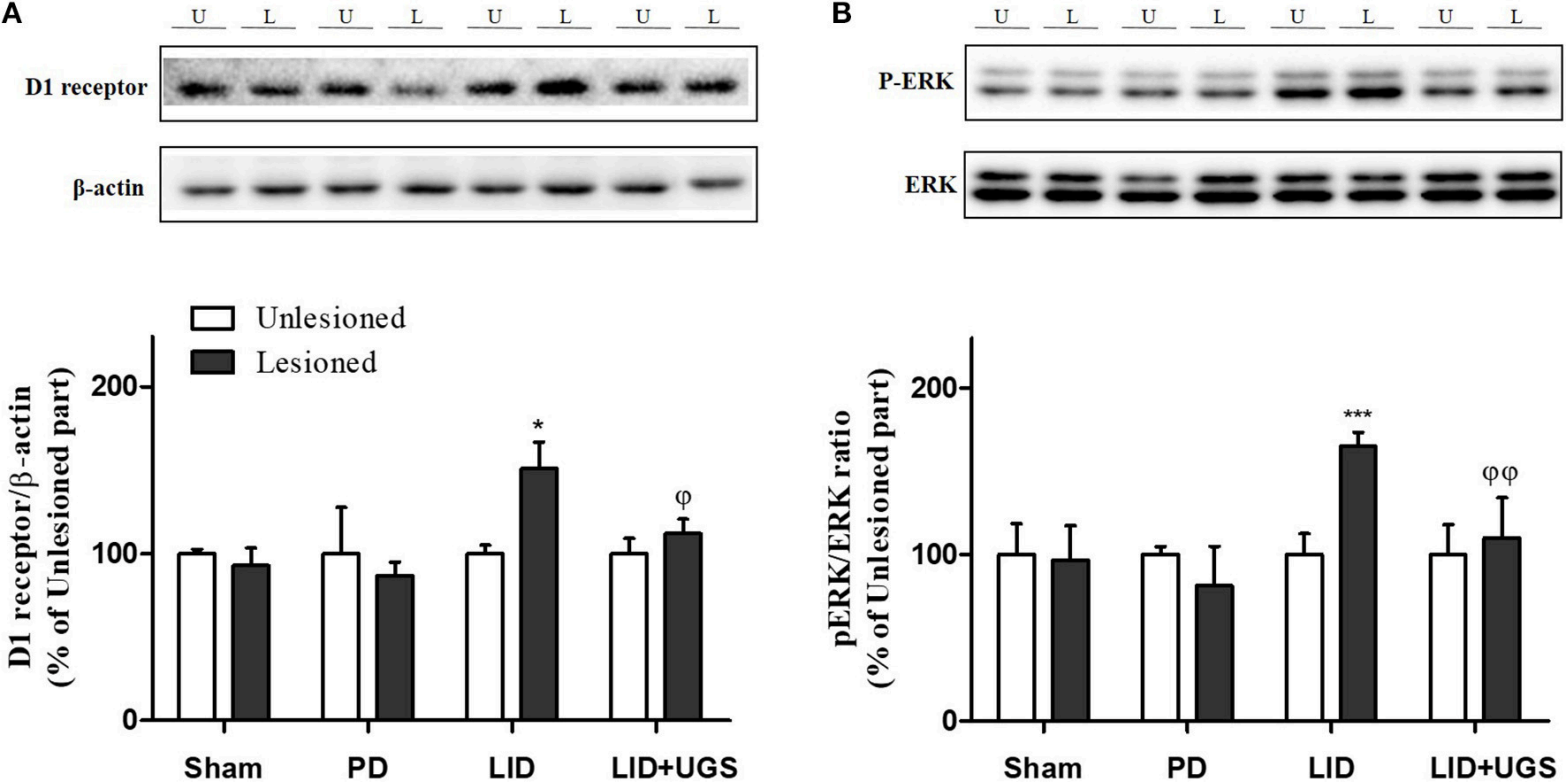
UGS reduced the expression of D1R and the phosphorylation of ERK after development of LID. Representative band images and the quantification data of D1R **(A)** and p-ERK **(B)** for western blot analysis. Values of quantification data are given as the mean ± S.E.M. (*N* = 6). The significance of differences was analyzed by one-way ANOVA followed by Bonferroni's Multiple Comparison Test: ^*^p < 0.05 and ^***^p < 0.001; mean values were significantly different from the unlesioned part of each group. ^ϕ^*p* < 0.05 and ^ϕϕ^*p* < 0.01; mean values were significantly different from the LID group.

Chronic administration of L-dopa leads to persistent and intermittent hyperactivation of the cAMP signaling cascade, which regulates several downstream effector targets such as ΔFosB and c-fos, responsible for the control of excitability of striatal neurons (Feyder et al., 2011). In the current study, western blot was performed to detect whether UGS normalized the L-dopa-induced overexpression of D1R and the phosphorylation of cAMP signaling-related protein, ERK, in the ST. The expression of D1R and p-ERK was significantly increased by L-dopa, while UGS reduced the overexpression in the lesioned section of the ST (Figure 8).

**Figure 9:**
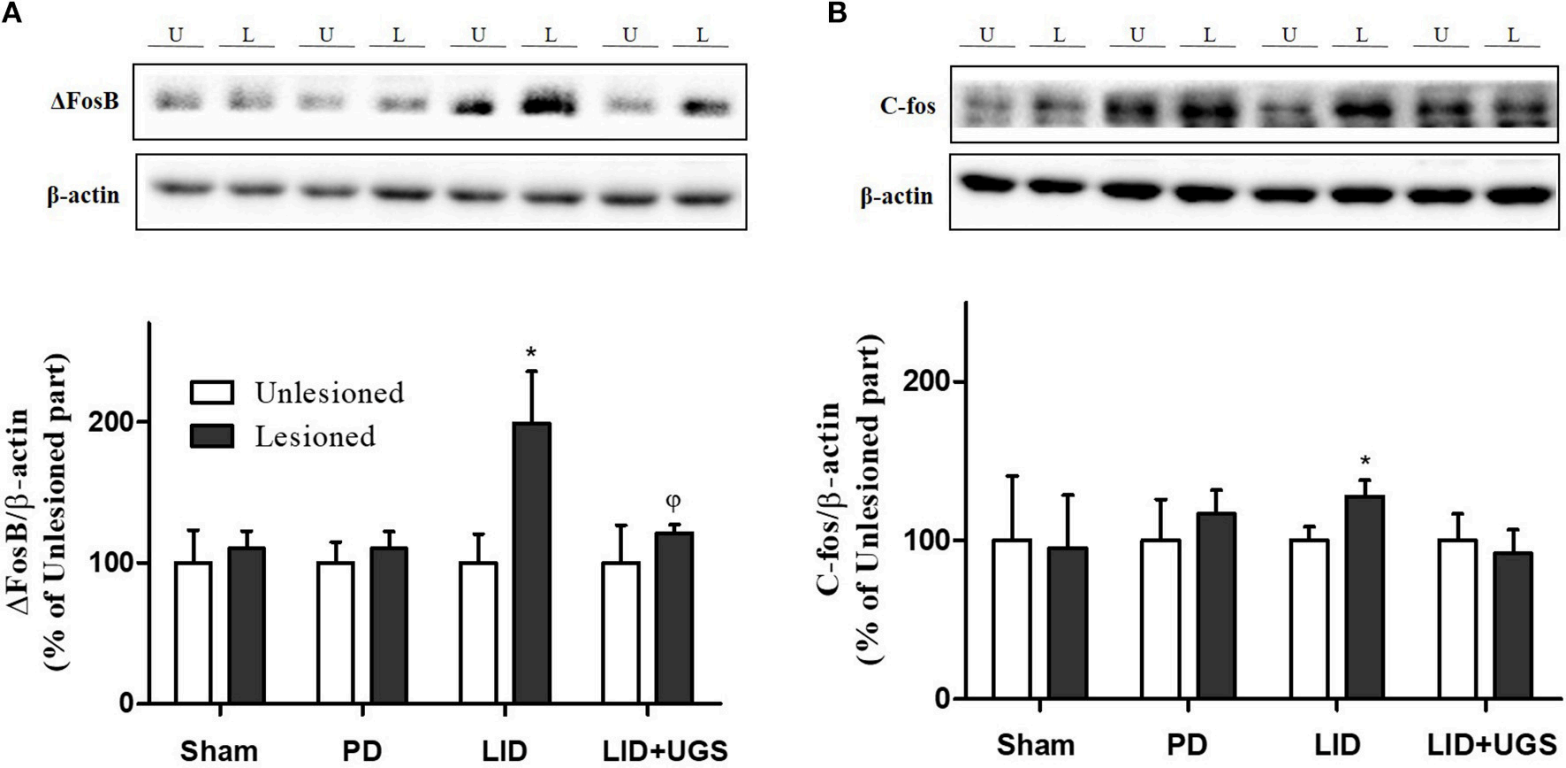
UGS inhibited the expression of ΔFosB and c-fos after development of LID. Representative band images and the quantification data of expression of ΔFosB **(A)** and c-fos **(B)** for western blot analysis. Values of quantification data are given as the mean ± S.E.M. (*N* = 6). The significance of differences was analyzed by one-way ANOVA followed by Bonferroni's Multiple Comparison Test: ^*^p < 0.05; mean values were significantly different from the unlesioned part of each group. ^ϕ^*p* < 0.05; mean values were significantly different from the LID group.

The proteins, ΔFosB and c-fos, are known as the downstream signaling proteins of ERK phosphorylation in the ST of the LID mouse model. The overexpression of ΔFosB and c-fos induced by D1R are implicated in various forms of synaptic plasticity, particularly in long-term potentiation (Chen et al., 2017). In the present study, the expression of transcriptional targets, such as ΔFosB and c-fos, was increased in the mouse ST of the LID group. UGS reduced the overexpression of ΔFosB and c-fos in the lesioned ST (Figure 9).

We confirmed that UGS inhibited dyskinetic movements induced by chronic administration of L-dopa in the 6-OHDA-injected PD mouse model. In addition, we found that UGS suppressed the D1R-ERK signaling pathway in the ST of the LID model.

## Discussion

In the current study, we investigated whether (1) the combined treatment of UGS with L-dopa attenuates motor functions in 6-OHDA-induced parkinsonian mice compared to L-dopa single treatment and whether (2) UGS ameliorates dyskinesia in response to chronic administration of L-dopa. This study clearly revealed that UGS increased the effects of L-dopa on motor dysfunction and inhibited L-dopa-induced dyskinetic movements. The effect of UGS on the survival of dopaminergic neurons in the SNpc and ST of 6-OHDA-induced parkinsonian mice and a reduction in D1R, p-ERK, ΔFosB, and c-fos levels in the ST of the LID mice were observed.

The unilaterally-lesioned 6-OHDA rodent model is the primary model used to reflect parkinsonian motor dysfunctions and LID; this surgical mouse model is referred to as “hemiparkinsonian syndrome.” The model is associated with complete dopamine depletion and hypersensitivity of lesioned, striatal dopamine receptors with lateralized behavior disorders of the forelimb contralateral to the lesion (Amalric et al., 1995). Upon chronic L-dopa treatment, 6-OHDA-lesioned mice showed AIM, and the biochemical mechanisms underlying the pathophysiology of LID are consistent with PD patients (Tronci and Francardo, 2017). Although the MPTP mouse model has been previously reported to replicate LID, it presents several drawbacks such as inconsistency in dyskinetic subtypes (hyperactive running and jumping) with clinical features of LID and variable degrees of behavioral and biochemical impairments (Nicholas, 2007). Therefore, the model used in this study is suitable for representing the clinical features of patients with PD and LID.

Since L-dopa is the precursor of dopamine, its effects merely supplement dopamine deficiency. In addition, the excessive supply of dopamine causes oxidative stress termed, auto-oxidation. Serra et al. (2000) found that the dopaminergic neuronal death by auto-oxidation of exogenous L-dopa occurs in both PC12 cells and the ST of rats. Administering L-dopa accelerates the progression of PD while temporarily mitigating symptoms of the disease. In the current study, UGS was selected for treatment along with L-dopa as it is one of the common drugs used to treat PD in East Asia and its mechanisms differ from those of L-dopa; the anti-parkinsonian effects of UGS are focused on neuroprotection and regulation of neurotransmissions (Doo et al., 2010; Kawanabe et al., 2010). This study revealed that combined treatment of UGS and L-dopa reduced the rigidity in 6-OHDA-lesioned mice compared to L-dopa alone and decreased dopaminergic neuronal damage in the nigrostriatal pathway as shown in Figure 2. Increased in the number of rotations upon combined treatment of UGS and L-dopa was also observed. Since dopamine depletion induces contralateral rotations in hemi-parkinsonian rodents, these results indicate that UGS influences the survival of dopaminergic neurons. Taken together, the behavioral manifestations after combined treatment seem to derived from the neuroprotective effects of UGS.

In the striatal projection neurons, the activation of D1R directly controls the excitatory signaling, whereas the activation of D2R indirectly regulates the excitatory signaling, which suppresses motor effects (Pavon et al., 2006). The expression of D1R is increased in response to the administration of L-dopa; therefore, its chronic use causes hypersensitivity or hyperactivation of D1R. In the current study, it was confirmed that the regulatory effects of UGS on D1R-related signaling are enhanced by L-dopa, which subsequently activates D2R in the ST.

Similarly, the overexpression of D1R in the dopamine-depleted ST of LID mice was observed. Pavon et al. (2006) and Fieblinger et al. (2014) have reported that overexpressing D1R results in an increase in related proteins while Feyder et al. (2016) reported that changes in mitogen-activated protein kinase signaling pathways are associated with increased D1R activation. Phosphorylation of ERK has also been shown to be involved in synaptic plasticity leading to long-term potentiation, which regulates the state of normal or abnormal form of motor learning. The subsequent long-lasting overexpression of ΔFosB and c-fos induced by p-ERK may also mediate long-term potentiation in the dopamine-depleted ST (Pavon et al., 2006). It has been suggested that hyperactivation in the glutamatergic corticostriatal pathway is related to the development of dyskinesia. We confirmed increased ΔFosB and c-fos levels induced by p-ERK in the lesioned ST with chronic L-dopa treatment. In addition, UGS normalizing the increase may be related to the amelioration of D1R expression and dopamine depletion.

In the present study, we investigated the interaction between UGS and L-dopa over time using AIMs assessment. The results indicated that there was an interaction between UGS and L-dopa with signs of dyskinetic symptoms; however, there was no effect on the drug's prolonged duration. In previous study, Ishida et al. (2016) reported that UGS prolonged the effectiveness of L-dopa via inhibiting COMT. In addition, they evaluated the effectiveness of L-dopa with the AIMs test and showed the prolonged duration of L-dopa when UGS was administered in rats. This is rather inconsistent with our results as duration was unaffected. In the previous study, the assessment was conducted on day 15 after L-dopa injection with a single injection of UGS; however, in this study, the interaction was estimated to take place on day 10 of UGS administration with chronic L-dopa. The differences between the two studies may be a result of the difference in the period of administration; however, further studies would be required to confirm.

D1R and D2R expression is also closely related to the activation of other receptors, such as metabotropic glutamate (mGlu) receptor or N-methyl-D-aspartate receptor in L-dopa-induced excitability. These receptors induce excessive Ca^2+^ influx and influence D1R activation while suppressing D2R activation (Morin et al., 2016). Among them, the mGlu5R and A2a-adrenoceptor interact with D2R, which regulates Akt/glycogen synthase kinase 3 beta and ERK signaling (Fieblinger et al., 2014; Zhang et al., 2016). It has been reported that UGS regulates adrenergic receptor activation. Nakagawa et al. (2012) suggested that the components in UGS inhibit morphine tolerance and physical dependence via suppressing A2a-adrenoceptor. It can be inferred that combining UGS with L-dopa normalizes both D1R and D2R via regulating the A2a-adrenoceptor; however, further studies on the mechanisms would be required to confirm.

In summary, UGS attenuated PD-like motor dysfunctions when treated with L-dopa via reduction of dopaminergic neuronal loss and normalization of D1R and D2R in 6-OHDA-injected mice. Moreover, UGS ameliorates dyskinetic movements induced by chronic administration of L-dopa via suppressing D1R-related signaling. These results suggest that UGS could be a promising agent for combination treatment with L-dopa for PD patients.

## Author Contributions

EH and MO designed and coordinated this study. EH, JC, and YS performed the experiments and analyzed the data. EH and MO wrote the manuscript. All the authors participated in discussion of the results and reviewed the final draft.

### Conflict of Interest Statement

The authors declare that the research was conducted in the absence of any commercial or financial relationships that could be construed as a potential conflict of interest.
